# Longitudinal Forecasting of Retinal Structure and Function Using a Multimodal StyleGAN-Based Architecture

**DOI:** 10.3390/bioengineering13020149

**Published:** 2026-01-28

**Authors:** Arunodhayan Sampathkumar, Danny Kowerko

**Affiliations:** Faculty Computer Science, Chemnitz University of Technology, 09111 Chemnitz, Germany; arunodhayan.sampath-kumar@cs.tu-chemnitz.de

**Keywords:** generative adversarial networks, GAN, OCT imaging, visual acuity forecasting, logMAR, ophthalmology, StyleGAN, multimodal forecasting, diabetic retinopathy

## Abstract

Generative Adversarial Networks (GANs) have emerged as powerful tools for medical image synthesis and clinical outcome prediction. In ophthalmology, accurate forecasting of Optical Coherence Tomography (OCT) images and best-corrected visual acuity (BCVA) values can significantly enhance patient monitoring and personalized treatment planning. We introduce a multimodal GAN inspired by the StyleGAN architecture, featuring super-resolution modules, a multi-scale patch discriminator, and temporal attention mechanisms. To predict logMAR values, a hybrid deep–shallow LSTM model was jointly trained alongside the image pipeline. Synthesized scans were processed through an EfficientNet-based classifier to predict 16 retinal biomarkers. To ensure subject independence, we employed a 3-fold patient-level cross-validation strategy. The proposed multimodal GAN achieved an SSIM of 0.9264, an FID of 11.9, and a PSNR of 38.1 dB for OCT forecasting. The logMAR module delivered an MAE of 0.052, while the biomarker classifier attained a macro-F1 score of 0.81. Based on logMAR change forecasting, patients were further categorized into Winner, Stabilizer, and Loser outcome groups using a threshold of Δ=0.05, achieving an overall F1 score of 0.84. Our approach effectively forecasts retinal morphology and functional outcomes, providing valuable predictive insights for proactive clinical decision-making in retinal health management.

## 1. Introduction

Accurate forecasting of a patient’s future medical condition is a crucial aspect of personalized healthcare, aiding in early intervention and optimized treatment planning [1]. In particular, predicting a patient’s next clinical visit, including generating medical imaging scans, can enhance diagnostic precision and improve patient outcomes. Recent advancements in deep learning, particularly generative models and time-series forecasting techniques, have enabled the possibility of synthesizing future medical scans based on a patient’s historical imaging data. Table 1 presents a detailed synthesis of current research studies in retinal disease, highlighting the transition from traditional machine learning to advanced deep learning methodologies across classification, regression, and generative tasks for diagnosis and treatment monitoring.

Traditional forecasting models often rely on regression-based or classification-based approaches. While these methods provide useful insights, they may struggle to capture complex, non-linear patterns inherent in many real-world phenomena. Such limitations can impact predictive accuracy, particularly when dealing with dynamic systems that evolve over time [1]. Time-series-based methods, in contrast, analyze sequential data points—either discrete or continuous—to identify trends, patterns, and dependencies over time. By preserving temporal relationships within the data, these models provide a more comprehensive framework for forecasting and decision-making. Advanced deep learning techniques, such as recurrent neural networks (RNNs), long short-term memory (LSTM) networks, and transformer-based architectures, further enhance predictive capabilities by capturing dependencies across longitudinal data [2,3].

**Table 1:** Summary of prior retinal analysis studies on treatment response, disease progression, and generative modeling.

Paper Reference	Model	Dataset	Disease	Treatment/Target	Task
[4]	SVM, RF, MLP	1029 pts (CATT)	nAMD	Anti-VEGF response	Regression
[5]	RF, SVM, ELM	254 DME pts	DME	Injection response	Regression
[6]	LR + RF	281 eyes	DME	CRT prediction	Regression
[7]	Multitask DL	1279 eyes	GA	Lesion progression	Regression
[8]	Lasso, RF, Boost	738 eyes	nAMD	Visual outcome	Regression
[9]	Attention GAN	1684 images	nAMD	Post-treatment synthesis	Generative
[10]	pix2pixHD (GAN)	632 pre/post pairs	DME	Post-treatment OCT	Generative
[11]	SHENet (GAN)	383 OCT volumes	nAMD	Longitudinal OCT	Generative
[12]	MuMo-GAN	8196 fundus images	AMD	Disease progression	Generative

Using time-series forecasting, it is possible to predict future images based on past observations, enabling applications such as medical imaging progression analysis, weather pattern visualization, satellite image forecasting, and video frame prediction. These models help in identifying subtle changes over time, improving early anomaly detection and long-term planning. One of the most promising approaches in this area is TimeGAN, a generative adversarial network specifically designed for time-series data. Unlike traditional GANs, which focus on independent data distributions, TimeGAN preserves temporal dependencies while generating synthetic sequences that closely mimic real data. By leveraging both supervised and unsupervised learning components, TimeGAN can effectively learn the underlying dynamics of sequential data, making it particularly useful for forecasting images where continuity and consistency across time steps are crucial [13,14].

Several studies have implemented time-series GANs on medical imaging data to generate future scans, aiding in disease progression prediction. The Sequence-Aware Diffusion Model (SADM) [15] focuses on longitudinal medical image generation, while cardiac aging synthesis from cross-sectional data with conditional GANs addresses cardiac MRI scans. GRAPE [16] introduces a multimodal dataset of longitudinal visual field and fundus images for glaucoma management, and a deep learning system for predicting the time to progression of diabetic retinopathy leverages deep learning for retinal disease forecasting. These works collectively enhance predictive capabilities in brain MRI [17], cardiac MRI [18], and fundus imaging using longitudinal data. MT-DENet [19] utilizes a multitemporal cascaded graph evolution module to capture patient-specific lesion trends from sequential OCT scans, predicting post-therapy disease progression. By integrating a feature similarity prior constraint based on medical knowledge, the model ensures anatomically realistic retinal structures and achieves state-of-the-art accuracy in both image synthesis and lesion volume estimation.

In this research, we propose a novel approach to forecasting a patient’s follow-up scan by leveraging previous visit scans and associated clinical information. Unlike conventional prediction models that focus on classification or segmentation, our method aims to generate synthetic medical scans that accurately reflect anticipated disease progression. To achieve this, we developed MedTimeGAN, a deep generative model that integrates temporal 3D CNNs, StyleGAN blocks, super-resolution, and a multi-scale patch discriminator. Additionally, we include a deep–shallow LSTM module to jointly forecast scalar logMAR values. Once the next-visit scan is generated, it is further analyzed using a pretrained EfficientNet-based classification network to predict 16 clinically relevant retinal biomarkers, namely the following: atrophy/thinning of retinal layers, disruption of the ellipsoid zone (EZ), disorganization of the retinal inner layers (DRIL), intraretinal (IR) hemorrhages, intraretinal hyperreflective foci (IRHRF), partially attached vitreous face (PAVF), fully attached vitreous face (FAVF), preretinal tissue or hemorrhage, vitreous debris (VD), vitreomacular traction (VMT), diffuse retinal thickening/macular edema (DRT/ME), intraretinal fluid (IRF), subretinal fluid (SRF), disruption of the retinal pigment epithelium (RPE disruption), serous pigment epithelial detachment (Serous PED), and subretinal hyperreflective material (SHRM). By incorporating longitudinal patient data, our approach provides a robust and unified framework for clinical decision-making, offering insights into disease evolution and assisting healthcare professionals in personalized treatment planning.

## 2. Dataset

The Ophthalmic Labels for Investigating Visual Eye Semantics (OLIVES) dataset [20] is a comprehensive, multimodal ophthalmic resource curated for machine learning research in disease progression modeling, diagnosis, and treatment planning. It contains longitudinal data from 96 eyes (patients), followed over an average of 66 weeks with approximately 16 clinical visits per eye. The dataset integrates scalar clinical measurements—best-corrected visual acuity (BCVA) and central subfield thickness (CST)—alongside expert-annotated vector biomarkers, two-dimensional near-infrared (NIR) fundus images, and three-dimensional Optical Coherence Tomography (OCT) volumes.

Each OCT volume consists of 49 B-scans acquired across the macula and represents a full 3D retinal scan for a given visit. In addition, the dataset includes NIR fundus images acquired per visit, longitudinal clinical measurements, and disease-level labels (diabetic retinopathy or diabetic macular edema). A consolidated tabular summary of the most important parameters of the data set is provided in Table 2.

To support temporal analysis, BCVA values originally provided in ETDRS letters were converted to logarithm of the minimum angle of resolution (logMAR) units using the standard ETDRS conversion [21]:

logMAR=1.7−(0.02×BCVAletters),

where higher logMAR values indicate worse visual acuity. Together with CST, these scalar values form the basis for longitudinal functional assessment in this study.

The OLIVES dataset is publicly available for academic research at https://zenodo.org/records/7105232, accessed on 22 January 2026.

### Experimental Data Usage and Design

The OLIVES dataset was used under two distinct experimental settings, corresponding to the two result tables reported in this study. Each setting addresses a different clinical objective and therefore employs a dedicated subset of the data and evaluation protocol.

**(1) GAN-based temporal modeling.** For generative temporal modeling experiments, only the TREX DMEcohort was used, comprising 47 unique patients with longitudinal follow-up. All available visits per patient were retained to capture disease progression dynamics. Each OCT visit consisted of 49 B-scans, which were used in their entirety to represent the full macular volume.

A custom PyTorch-v2.6 Dataset class was implemented to group OCT data by (Patient_ID, Visit), apply preprocessing and normalization, and construct visit-level tensors of shape. Missing or unreadable B-scans were replaced with zero-valued tensors to maintain consistent input dimensionality during training. A 3-fold cross-validation strategy was applied at the patient level, ensuring that all visits from a given patient were confined to a single fold and preventing identity leakage.

For qualitative evaluation and visual comparison against the ground truth, the central slice (25th B-scan) of each 49-slice OCT volume—corresponding to the foveal center—was used. This selection was motivated by a statistical analysis of the available ground-truth biomarker annotations at the first and last visits, which confirmed that biomarkers are consistently present within the central slice and the surrounding range of slices (20–30). Notably, at the 25th slice, no biomarker annotations were missing across all annotated visits, whereas missing annotations occurred primarily at more peripheral slices. From an anatomical and functional perspective, the fovea represents the retinal region responsible for the highest visual acuity and is central to clinically relevant measures such as best-corrected visual acuity and central subfield thickness; its structure and functional importance have been quantitatively characterized using OCT in prior work [22]. A quantitative evaluation of the GAN-based temporal prediction models is reported in Table 5.

**(2) Biomarker classification.** For biomarker classification experiments, OCT B-scans from both the TREX DME (47 patients) and Prime_FULL (40 patients) cohorts were used. In this setting, only the **first and last visits** per eye were considered, as expert biomarker annotations are available exclusively at these timepoints. Classification was performed at the slice level using individual B-scans with corresponding biomarker labels. Classification performance is reported in Table 6.

## 3. Multimodal Architecture

**Figure 1:**
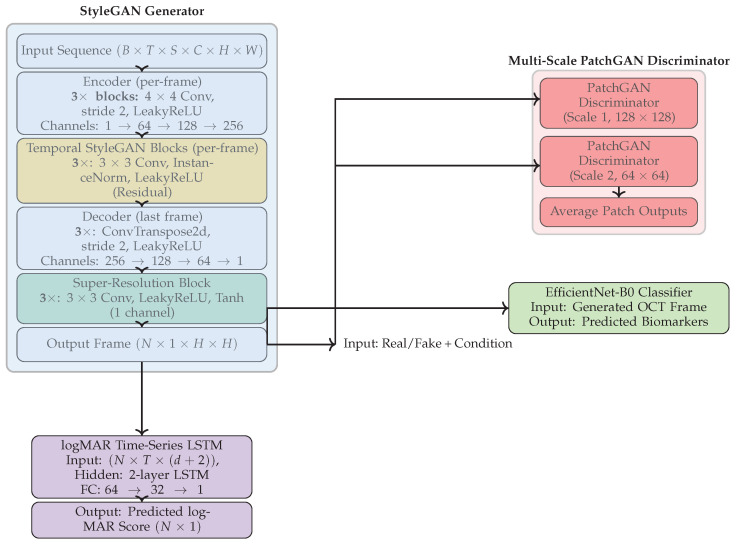
Overview of the proposed multimodal framework integrating a StyleGAN-based generator, a multi-scale PatchGAN discriminator, an EfficientNet-B0 classifier for biomarker prediction, and a temporal DeepShallow LSTM model for logMAR forecasting. The model jointly forecasts high-resolution future OCT frames, visual acuity outcomes, and biomarker status from historical time-series input.

Our proposed framework is designed for time-series image forecasting, super-resolution, biomarker prediction, and clinical outcome forecasting. It draws inspiration from StyleGAN and modern adversarial learning techniques. The architecture consists of four jointly trained components: (1) a StyleGAN-based generator for synthesizing future OCT frames, (2) a multi-scale PatchGAN discriminator for enforcing spatial realism, (3) an EfficientNet-B0 classifier for predicting disease-relevant biomarkers from generated OCT frames, and (4) a DeepShallow LSTM module for logMAR from historical data. An overview is illustrated in Figure 1.

### 3.1. StyleGAN Generator

The proposed generator architecture, *G*, is designed to synthesize foveal B-scans by integrating spatiotemporal features from historical longitudinal OCT volumes. The model leverages a style-based refinement mechanism to handle the inherent noise and intensity variations across different imaging sessions.

Spatial–Temporal Encoding

The input to the generator is a 6D tensor $X\in R^{B\times T\times S\times C\times H\times W}$, where *B* is the batch size, *T* is the number of longitudinal visits, S=49 represents the number of B-scans per volume, c=1 is the channel, and the spatial dimensions are H × W. Each slice is first processed by a spatial encoder E consisting of three strided convolutional layers. For a single slice $x_{t,s}$ at visit *t* and slice index *s*, the initial feature extraction is defined as follows:

(1) $$z_{t,s}=\sigma \left(\mathrm{Conv}\left(x_{t,s}\right)\right)$$

where σ denotes the LeakyReLU activation function. To account for disease progression over time, the model performs a temporal integration of features across all historical visits. The global temporal feature map $Z_{temp}$ is computed via mean aggregation:

(2) $$Z_{temp}=\frac{1}{T}\sum_{t=1}^{T}V_{t}$$

where $V_{t}$ represents the aggregated volumetric feature for a specific visit.

Contextual Modeling with StyleGAN Blocks

To refine the latent anatomical representations, we employ specialized blocks inspired by the StyleGAN architecture. Each block utilizes Instance Normalization (IN) to disentangle the anatomical “content” from the imaging “style” (e.g., gain artifacts or noise). The transformation for a feature map *h* within these blocks is formulated as follows:

(3) $$\hat{h}=\sigma \left(\mathrm{IN}\left(\mathrm{Conv}\left(h\right)\right)+h\right)$$

We implement a *foveal-centric contextual fusion* strategy within each visit. Given that the 25th slice (s=24 in 0-based indexing) typically represents the foveal center, we define the visit-wise feature $V_{t}$ as follows:

(4) $$V_{t}=z_{t,\mathrm{fovea}}+\alpha \left(\frac{1}{S-1}\sum_{s\neq \mathrm{fovea}}z_{t,s}\right)$$

where α=0.3 is a scaling coefficient. This allows the network to prioritize the fine structural details of the fovea while maintaining global volumetric consistency informed by the peripheral slices.

Decoding and Super-Resolution

The decoder *D* maps the fused spatiotemporal features back to the pixel-intensity manifold. This is achieved through a sequence of transposed convolutions that progressively recover spatial resolution to an intermediate representation of 128×128. The final output layer of the decoder consists of a 3×3 convolution followed by a Tanh activation to ensure the predicted intensities are bounded within [−1,1]:

(5) $${\hat{y}}_{128}=\mathrm{Tan}h\left({\mathrm{Conv}}_{3\times 3}\left(D\left(Z_{temp}\right)\right)\right)$$

### 3.2. Multi-Scale PatchGAN Discriminator

To assess the realism of generated frames, we employ a multi-scale PatchGAN discriminator operating at two spatial resolutions:

Patch-Based Supervision

Each discriminator receives a concatenation of the historical input frame and either the real or generated next frame. At full scale (128×128) and half scale (64×64), patch-level discrimination jointly enforces global structural consistency and fine-grained local realism. In particular, the smaller patch size is effective in constraining thin retinal layers and small anatomical structures, while the larger patch size promotes coherent global retinal geometry.

Adversarial Optimization

The outputs from each scale are averaged to form the total adversarial loss

(6) $$L_{\mathrm{adv}}=\frac{1}{2}\left(L_{D_{1}}+L_{D_{2}}\right)$$

pushing the generator to produce visually plausible and anatomically accurate frames.

### 3.3. logMAR Forecasting via DeepShallow LSTM

To jointly predict clinical outcomes, we integrate a DeepShallow LSTM model that consumes both structured clinical data and visual embeddings to forecast future logMAR scores.

Multimodal Input Representation

The input to the LSTM model is a multivariate time series composed of three elements:

(1) Historical best-corrected visual acuity (logMAR);
(2) Central subfield thickness (CST);
(3) Visual latent features extracted from the generator’s encoder.

These components are concatenated per visit, forming the multimodal input tensor:

$$Z\in R^{N\times T\times (2+d)},$$

where *T* is the number of historical visits, the scalar features (logMAR, CST) contribute two dimensions, and *d* denotes the encoder’s latent embedding dimension.

Temporal Regression

A two-layer LSTM with a hidden size of 64 captures disease progression patterns across visits. The final hidden state is passed through a fully connected regressor as follows:

LSTMoutput→FClayers:64→32→1,

This generates the predicted logMAR value at the next timepoint.

This multimodal formulation enables the model to learn complex interactions between anatomical features, prior visual outcomes, and imaging-based representations.

### 3.4. Training Objective

The full model is optimized end-to-end using a composite objective that integrates adversarial learning, reconstruction fidelity, spatial smoothness, and clinical regression accuracy:

(7) $$L_{\mathrm{total}}=\lambda_{\mathrm{adv}}L_{\mathrm{adv}}+\lambda_{\mathrm{rec}}L_{\mathrm{recon}}+\lambda_{\mathrm{tv}}L_{\mathrm{tv}}+\lambda_{\mathrm{reg}}L_{\mathrm{logMAR}}$$

where

- $L_{\mathrm{adv}}$: Adversarial loss from multi-scale PatchGAN discriminators;
- $L_{\mathrm{recon}}$: Pixel-wise or perceptual reconstruction loss (e.g., $\ell_{1}$ or VGG loss) between predicted and ground-truth frames;
- $L_{\mathrm{tv}}$: Total variation loss that encourages smoothness and reduces artifacts in the generated image;
- $L_{\mathrm{logMAR}}$: Clinical regression loss that penalizes errors in logMAR forecast. It is defined as follows:
  (8) $$L_{\mathrm{logMAR}}=\frac{1}{N}\sum_{i=1}^{N}{\left({\hat{y}}_{\mathrm{logMAR}}^{\left(i\right)}-y_{\mathrm{logMAR}}^{\left(i\right)}\right)}^{2}$$
  where ${\hat{y}}_{\mathrm{logMAR}}^{\left(i\right)}$ is the predicted score and $y_{\mathrm{logMAR}}^{\left(i\right)}$ is the ground truth.

The weights λ are tuned empirically to balance visual fidelity and clinical outcome accuracy. In our experiments, we found that $\lambda_{\mathrm{adv}}=1$, $\lambda_{\mathrm{rec}}=10$, $\lambda_{\mathrm{tv}}=0.1$, and $\lambda_{\mathrm{reg}}=5$ yielded optimal trade-offs across validation metrics.

### 3.5. Temporal Modeling Strategy

To model disease progression over time and forecast both future OCT scans and logMAR values, we adopt a sliding window strategy across longitudinal visit data for each patient. The dataset provides up to 10 timepoints per eye, labeled from $v_{1}$ to $v_{10}$. From these, we construct temporal training samples using overlapping pairs and triplets of historical visits to predict subsequent visits.

For example, using two-visit inputs, we form training samples such as $\left(v_{1},v_{2}\right)\to v_{3}$, $\left(v_{2},v_{3}\right)\to v_{4}$, and so on up to $\left(v_{8},v_{9}\right)\to v_{10}$. Similarly, for three-visit inputs, we generate sequences such as $\left(v_{1},v_{2},v_{3}\right)\to v_{4}$, …, $\left(v_{7},v_{8},v_{9}\right)\to v_{10}$. These samples are stacked across the dataset to form a rich set of temporally coherent training examples.

Each input window includes full 3D OCT volumes from 49 B-scans per visit, resized to a resolution of 256×256. These volumes are processed by the generator to synthesize the future visit’s B-scans. For logMAR prediction, a separate LSTM-based regression head consumes the same historical window to forecast the scalar logMAR value corresponding to the predicted scan.

During training, we ensure that the prediction target $v_{t}$ is never included in the input window to avoid temporal leakage. The model is trained in a teacher-forcing mode, where each prediction is made independently based on previous ground-truth visits. This design supports both single-step forecasting during training and autoregressive inference during evaluation.

To improve robustness, we aggregate training samples from all available patient trajectories, padding sequences where fewer than 10 visits exist. The total training set thus captures a wide range of disease stages, injection events, and recovery patterns across multiple time horizons.

### 3.6. Training and Loss Functions

The model is trained within a conditional adversarial learning framework, where the generator and discriminator are jointly optimized to produce high-fidelity, perceptually realistic future frames from spatiotemporal image sequences. Training alternates between updating the generator and discriminator, enabling adversarial feedback to continually refine output realism and anatomical accuracy.

#### 3.6.1. Generator Objectives

The generator is trained using a composite objective that combines adversarial, pixel-wise reconstruction, perceptual, and structural similarity losses. The adversarial loss encourages the generator to produce outputs that the discriminator classifies as real, while pixel-level accuracy is enforced via an L1 reconstruction loss. Perceptual similarity is promoted using a VGG16-based feature loss, and structural consistency is preserved through an SSIM loss.

The adversarial loss is implemented using a binary cross-entropy loss with logits:

(9) $$L_{\mathrm{adv}}=-E_{\hat{x}}\left[\log\sigma \left(D(x,\hat{x})\right)\right],$$

where $\hat{x}$ denotes the generated image, *x* is the ground-truth image, D(·) represents the discriminator output logits, and σ(·) is the sigmoid function.

The pixel-wise reconstruction loss is defined as the L1 distance between the generated and real images:

(10) $$L_{\mathrm{pixel}}={∥\hat{x}-x∥}_{1}.$$

To improve perceptual fidelity, a perceptual loss is computed as the mean squared error between VGG16 feature representations of the generated and ground-truth images:

(11) $$L_{\mathrm{perc}}={∥\phi \left(\hat{x}\right)-\phi \left(x\right)∥}_{2}^{2},$$

where ϕ(·) denotes activations from a pretrained VGG16 network (layers up to conv3_3). Grayscale inputs are replicated across three channels prior to feature extraction.

Structural similarity is enforced using an SSIM-based loss:

(12) $$L_{\mathrm{SSIM}}=1-\mathrm{SSIM}\left(\hat{x},x\right),$$

where SSIM is computed with a data range of L=2.0, consistent with the normalized image intensity range.

The final generator objective is given by the following:

(13) $$L_{G}=L_{\mathrm{adv}}+\lambda_{1}L_{\mathrm{pixel}}+\lambda_{2}L_{\mathrm{perc}}+\lambda_{3}L_{\mathrm{SSIM}},$$

with $\lambda_{1}=5$, $\lambda_{2}=0.5$, and $\lambda_{3}=10$.

#### 3.6.2. Discriminator Training

The discriminator is trained using a composite loss function that integrates binary cross-entropy (BCE) with a Wasserstein distance metric and a gradient penalty term. This hybrid approach leverages the classification accuracy of BCE while utilizing the Wasserstein distance to provide a smoother gradient landscape, which helps the generator learn more effectively even when the distributions are far apart.

To further improve stability and enforce smoothness in the discriminator’s output, a gradient penalty ($L_{\mathrm{GP}}$) is added. This regularization encourages the gradients of the discriminator with respect to its input to have unit norm, which plays a critical role in mitigating mode collapse and ensuring consistent convergence throughout the training process.

The total discriminator loss $L_{D}$ is formulated as follows:

(14) $$L_{D}=\frac{1}{2}\left(L_{\mathrm{BCE},\mathrm{real}}+L_{\mathrm{BCE},\mathrm{fake}}\right)+\gamma \left(E_{\tilde{x}∼P_{g}}\left[D\left(\tilde{x}\right)\right]-E_{x∼P_{r}}\left[D\left(x\right)\right]\right)+\lambda_{\mathrm{GP}}L_{\mathrm{GP}}$$

where γ is the weight for the Wasserstein component and $\lambda_{\mathrm{GP}}$ is the gradient penalty weight, set to 10 in our implementation. The gradient penalty is calculated based on the norm of the gradients with respect to interpolated samples $\hat{x}$:

(15) $$L_{\mathrm{GP}}=E_{\hat{x}∼P_{\hat{x}}}\left[{\left(∥\nabla_{\hat{x}}D\left(\hat{x}\right){∥}_{2}-1\right)}^{2}\right]$$

where $\hat{x}$ is a random interpolation between real and synthesized image pairs.

#### 3.6.3. Biomarker Classification Training

In addition to forecasting OCT scans and logMAR values, the generated OCT frames are analyzed using a pretrained EfficientNet-B0 classifier to predict 16 clinically relevant retinal biomarkers. The classifier is trained on the OLIVES dataset, which provides expert-annotated labels for biomarker presence across patient visits. The predicted biomarkers include the following: atrophy or thinning of retinal layers, disruption of the ellipsoid zone (EZ), disorganization of the retinal inner layers (DRIL), intraretinal hemorrhages (IR), intraretinal hyperreflective foci (IRHRF), partially attached vitreous face (PAVF), fully attached vitreous face (FAVF), preretinal tissue or hemorrhage, vitreous debris (VD), vitreomacular traction (VMT), diffuse retinal thickening or macular edema (DRT/ME), intraretinal fluid (IRF), subretinal fluid (SRF), retinal pigment epithelium (RPE) disruption, serous pigment epithelial detachment (Serous PED), and subretinal hyperreflective material (SHRM).

To address the substantial class imbalance among biomarkers, the classifier is optimized using focal loss, which down-weights easy negatives and emphasizes harder, underrepresented cases. The focal loss is defined as follows:

(16) $$L_{\mathrm{focal}}=-\alpha_{t}{\left(1-p_{t}\right)}^{\gamma }\log\left(p_{t}\right),$$

where $p_{t}$ denotes the predicted probability of the true class, $\alpha_{t}$ is a class-balancing factor, and γ is the focusing parameter, set to 2 in our experiments.

The classifier is trained using the Adam optimizer with an initial learning rate of $1\times 10^{-3}$, a batch size of 32, and a weight decay of $1\times 10^{-5}$. A cosine learning rate scheduler with warmup is employed to stabilize early optimization, and training is performed for up to 50 epochs with early stopping applied when the validation loss does not improve for 10 consecutive epochs. To improve generalization and robustness, data augmentation strategies including random flips, rotations, brightness adjustments, and MixUp are applied during training.

#### 3.6.4. Training Strategy

Generator and Discriminator Training

The generator and discriminator are optimized using the Adam optimizer, with learning rates of $2\times 10^{-4}$ and $1\times 10^{-4}$, respectively. Both networks are trained with a batch size of 8 and a weight decay of $1\times 10^{-6}$. Training is performed for up to 350 epochs, with early stopping applied if the validation loss does not improve for 10 consecutive epochs. A cosine annealing learning rate scheduler is employed to promote stable convergence and avoid premature stagnation.

All models are trained using NVIDIA H100 GPUs provided by the National High-Performance Computing Center (NHR) at TU Dresden.

#### 3.6.5. Evaluation Metrics

To quantitatively assess performance, we compute the following metrics during training:

- **Peak Signal-to-Noise Ratio (PSNR):** Captures the signal fidelity between generated and ground-truth OCT outputs. A higher PSNR correlates with a lower pixel-wise reconstruction error.
- **Structural Similarity Index Measure (SSIM):** Evaluates structural integrity, contrast preservation, and perceptual quality. SSIM is particularly sensitive to spatial distortions and is more aligned with human visual perception than pixel-wise metrics.
- **F1 Score (Macro-Averaged):** Used to evaluate biomarker classification performance across the 16 predicted categories. This metric balances precision and recall and ensures that less frequent biomarkers are weighted equally in the overall evaluation.

## 4. Results

This section comprehensively evaluates the proposed multimodal forecasting framework across anatomical, functional, and clinical dimensions. Experiments were performed on longitudinal OCT sequences with corresponding visual acuity (logMAR) measurements. The objective was to assess the model’s capability to forecast future retinal morphology and functional trends from prior visits.

Results are organized into five parts: (1) qualitative visualization of OCT forecasts; (2) quantitative analysis of logMAR trend prediction using the Winner–Stabilizer–Loser framework; (3) model comparison and ablation analysis of generators, discriminators, and loss functions; (4) multimodal biomarker classification from predicted OCTs; and (5) benchmarking against prior longitudinal forecasting methods.

Overall, the proposed model demonstrates three key properties:

- **High anatomical fidelity**, with predicted OCT frames achieving SSIM up to **0.93** and PSNR exceeding **38 dB**;
- **Clinically consistent functional forecasting**, with logMAR errors (MAE = **0.052**) well within the accepted ±0.1 clinical tolerance;
- **Trend-awareness**, as the model accurately identifies whether a patient’s visual function is improving, stable, or declining across visits.

These results collectively establish that the proposed multimodal GAN learns both spatial structure and temporal dynamics, producing anatomically realistic and clinically interpretable forecasts suitable for longitudinal disease monitoring.

### 4.1. Qualitative OCT Forecasting Performance

**Figure 2:**
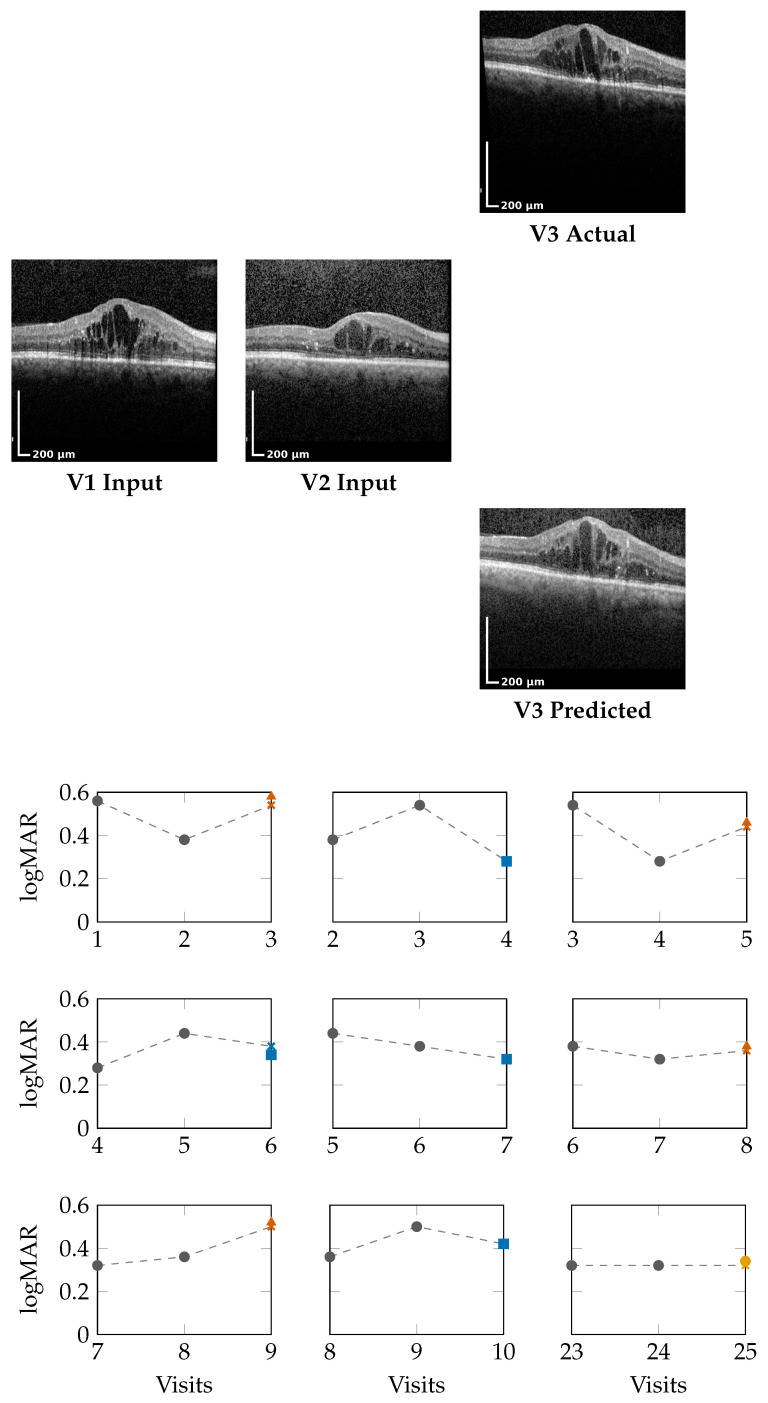
Patient 232 class encoding: Winner (Δ≥−0.05), Stabilizer (|Δ|<0.05), Loser (Δ≤0.05). Prediction crosses match class color.

**Figure 3:**
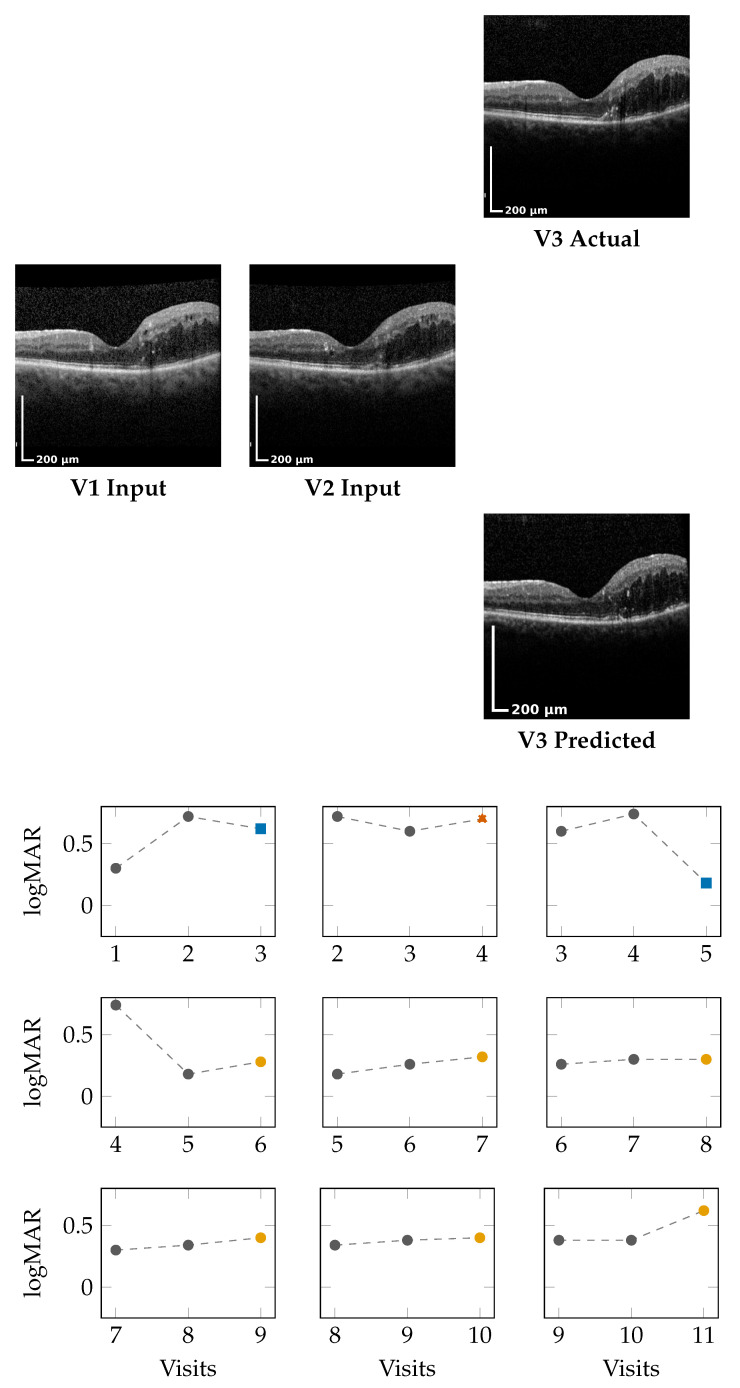
Patient 217 logMAR trajectories using the same colorblind-safe classes: Winner, Stabilizer, Loser. Prediction crosses use matching class colors.

Figure 2 (top) and Figure 3 (top) present qualitative OCT forecasting results for two representative patients (IDs 232 and 217). In both examples, the model receives two consecutive OCT scans (V1 and V2) and predicts the subsequent visit (V3). The predicted frames exhibit high visual fidelity and strong anatomical consistency with the ground truth. Specifically, the forecasts demonstrate the following:

- **Preservation of retinal layer continuity**, particularly around the foveal pit and outer retinal boundaries;
- **Accurate modeling of macular thickness and edema progression**, critical biomarkers for diabetic macular edema (DME) and age-related macular degeneration (AMD);
- **Retention of fine microstructural features**, facilitated by the super-resolution and StyleGAN-inspired upsampling mechanisms in the generator network.

These results confirm that the proposed model effectively learns spatial and temporal correlations within the retinal morphology, capturing both local structural changes and global thickness variations across visits. Subtle pathological signatures, such as localized depressions and intraretinal cystic spaces, are preserved in the predicted frames, demonstrating the model’s capacity to generalize across disease stages and longitudinal follow-ups.

### 4.2. Forecasting Functional Outcome (logMAR)

The lower panels of Figure 2 and Figure 3 illustrate sliding window forecasts of logMAR trajectories for Patients 232 and 234. Each subplot represents a temporal triplet $\left(V_{t},V_{t+1}\to V_{t+2}\right)$, where

- Gray circles indicate ground-truth logMAR values at $V_{t}$ and $V_{t+1}$;
- Colored markers show the model’s forecast at $V_{t+2}$ using a colorblind-safe palette;
- Cross-shaped “×” markers indicate the ground-truth logMAR at $V_{t+2}$, colored with the same class color for direct comparison.

Predicted outcomes are categorized using the deviation Δ between prediction and the previous visit:

$$\mathrm{Delta}\mathrm{Calculation}:\;\Delta =V_{\mathrm{prev}}-V_{\mathrm{next}},\;V_{\mathrm{next}}\in \left\{V_{\mathrm{next},\mathrm{GT}},\;V_{\mathrm{next},\mathrm{Pred}}\right\}.$$

$$\mathrm{Outcome}\mathrm{Classification}:\;Class\left(\Delta \right)=\left\{\begin{array}{cc} \mathrm{Winner}, & \Delta \geq \delta ,\\ \mathrm{Stabilizer}, & -\delta <\Delta <\delta ,\\ \mathrm{Loser}, & \Delta \leq -\delta , \end{array}\right.\;\delta =0.05.$$

- **Winner** (Δ≥−0.05): predicted **improvement** in visual acuity (lower logMAR);
- **Stabilizer** (|Δ|<0.05): predicted **stable** visual acuity;
- **Loser** (Δ≤+0.05): predicted **deterioration** (higher logMAR).

**Patient 232 (Figure 2):** Across nine temporal windows, the model successfully tracks logMAR evolution and the overall disease trend.

- In the V1,V2 → V3 window, the model’s numerical estimate is slightly higher than the true logMAR deterioration (0.58 vs. 0.54), but it still correctly assigns the Loser class.
- In the V2,V3 → V4 window, the prediction matches the ground truth (0.28), indicating a Winner.
- In later visits, e.g., V5,V6 → V7, the prediction (0.32) equals GT (0.32), representing a Stabilizer.

This consistency across sequential visits highlights that the model not only produces accurate logMAR values but also respects the *directionality* of visual function changes, correctly identifying improvement, stability, or decline phases.

**Patient 217 (Figure 3):** For this patient, logMAR values remain in the positive range, indicating moderate visual impairment with temporal fluctuations across visits. Overall, the model demonstrates the ability to capture both improvement and stabilization patterns in a stepwise forecasting setting:

- In the early V1,V2 → V3 window, the forecast (0.62) closely follows the ground truth (0.60), resulting in a Winner outcome under the defined threshold.
- In the subsequent V2,V3 → V4 window, the predicted value (0.70) deviates beyond the stabilization margin from GT (0.60), corresponding to a Loser.
- From V3,V4 → V5, the model accurately captures a marked improvement, predicting 0.18 compared to a GT of 0.74, yielding a clear Winner.
- In later windows (e.g., V5,V6 → V7 and V6,V7 → V8), predictions remain within ±0.05 of the corresponding GT values, indicating consistent Stabilizer behavior.
- Towards the end of the sequence (V8,V9 → V10 and V9,V10 → V11), the model preserves stability, with forecasts closely aligned to GT, despite a rise in absolute logMAR values.

Across visits, predictions remain well within clinical tolerance thresholds (±0.05), demonstrating robustness even when logMAR reversals or subtle fluctuations occur.

**Summary:** The results demonstrate that the model does not merely perform pointwise regression but learns the **underlying temporal behavior of visual function**. The Winner–Stabilizer–Loser classification framework provides an interpretable means to assess prediction reliability and trend consistency:

- Winners correspond to predicted improvements, highlighting recovery tendencies;
- Stabilizers dominate across visits, showing that the model can maintain disease stability over time;
- Losers appear infrequently within the shown examples and usually represent small deviations within the noise margin of measurement error.

By correctly identifying the temporal direction of change, the model demonstrates strong potential for **trend-aware visual prognosis**, enabling longitudinal interpretation of patient recovery or decline.

### 4.3. Winner–Stabilizer–Loser Classification Across Thresholds

Table 3 presents the distribution of **Winner, Stabilizer, and Loser** outcomes for four patients (IDs 201, 203, 232, and 234) across varying tolerance thresholds (Δ=0.01–0.10). Lower δ values represent strict thresholds (minor deviations are penalized), while higher δ values allow greater clinical tolerance.

- **Patient 201:** Shows balanced outcomes at δ=0.01 but converges to entirely stable predictions by δ≥0.04, with no Losers remaining.
- **Patient 232:** Initially sensitive to small fluctuations (13 Losers at δ=0.01), yet transitions fully to Stabilizers beyond δ≥0.08, demonstrating consistency under realistic tolerances.
- **Patient 234:** Exhibits steady improvement; Losers diminish progressively, and all predictions become Stabilizers by δ≥0.06.
- **Patient 236:** Displays early variability but stabilizes completely by δ≥0.07, mirroring trends in Patient 232.

This trend-based interpretation highlights that the model adapts well across patients and tolerance levels, consistently maintaining temporal coherence in visual function forecasting.

### 4.4. Confusion Matrix Evaluation at Δ=0.05

**Figure 4:**
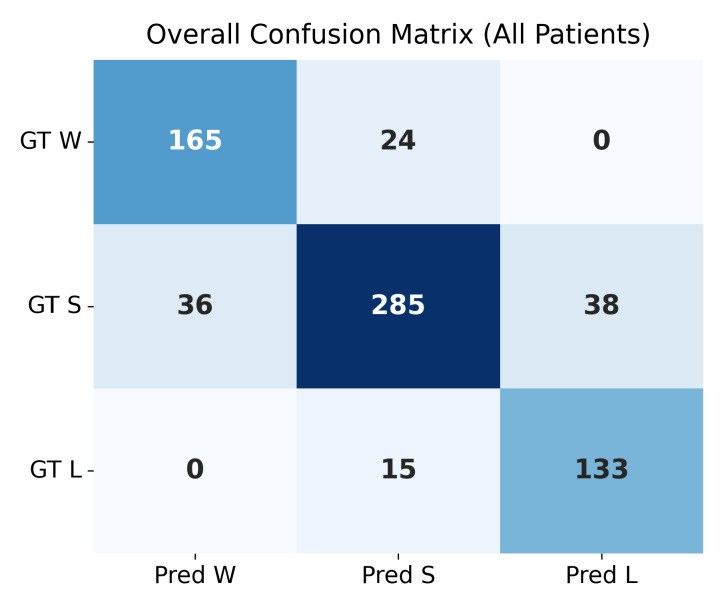
Overall confusion matrix across the full test set (20 patients). Performance metrics are provided in Table 4.

**Figure 5:**
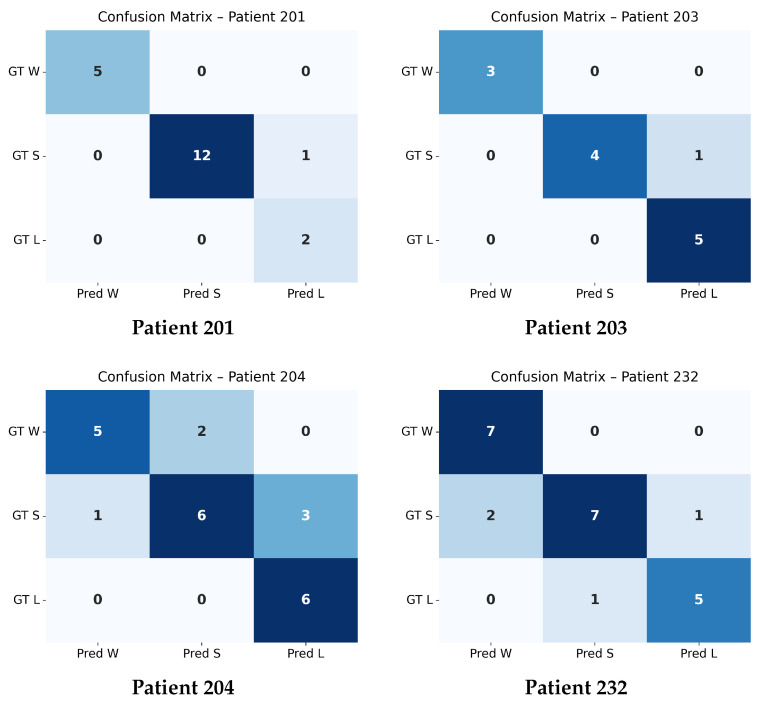
Individual confusion matrices for the four patients arranged in a 2 × 2 layout, computed using the classification threshold Δ=0.05. Performance metrics for each patient are reported in Table 4.

The confusion matrices shown in Figure 4 and Figure 5 summarize the agreement between the model’s predicted trajectory classes and the ground-truth delta-based labels for each patient. Each matrix compares three clinically relevant progression categories:

- **Winner (W):** Improvement beyond the negative threshold (Δ<−0.05);
- **Stabilizer (S):** Minimal change within the threshold range (−0.05≤Δ≤0.05);
- **Loser (L):** Worsening beyond the positive threshold (Δ>0.05).

Correct predictions appear along the diagonal of each matrix, whereas off-diagonal values represent misclassifications. A higher concentration of values along the diagonal therefore indicates stronger predictive accuracy. The overall confusion matrix exhibits a clear diagonal dominance, demonstrating that the model reliably differentiates between improving (W), stable (S), and worsening (L) trajectories across all patients.

Individual patient confusion matrices reveal patient-specific performance patterns. Patients 201, 203, and 232 show strong diagonal structures with minimal misclassification, indicating highly predictable progression patterns. Patient 204 displays more variability, with a larger number of S-to-L confusions, suggesting more complex or borderline changes around the threshold.

A detailed quantitative summary of precision, recall, and F1-scores for the overall dataset and each patient is provided in Table 4. These metrics reinforce the visual observations from the confusion matrices and confirm that the model performs consistently across most patients, with only minor deviations in more challenging cases.

### 4.5. Quantitative Results and Model Comparison

Table 5 presents a comprehensive evaluation of multiple generator–discriminator configurations, input sequence lengths, loss formulations, and training strategies for OCT time-series forecasting. Models with IDs 1–4 correspond to baseline configurations evaluated during the initial design phase. Specifically, ID 1 represents a transformer-based temporal model with positional encoding, ID 2 corresponds to a temporal CNN model combined with a super-resolution module, and IDs 3–4 denote early variants of **Generator 1** without StyleGAN-inspired feature modulation. Although transformer-based and other attention-driven temporal models are widely regarded as state of the art, they typically require large-scale datasets to generalize effectively. Given the moderate size of the OLIVES dataset and the limited temporal context available per subject, simpler temporal CNN-based architectures provided more stable optimization and better generalization in this setting. We further observe a gradual increase in SSIM for configurations predicting later visits or using longer temporal context. This behavior reflects reduced structural variability and smoother anatomical progression typically observed in longitudinal OCT data at later stages, where inter-visit changes become smaller and more predictable. Prior longitudinal OCT studies [23] have shown that the largest retinal structural changes occur early during disease progression or treatment, followed by more stable retinal morphology over time, which naturally favors higher structural similarity metrics such as SSIM.

All models were trained with a maximum of 350 epochs using identical optimization settings and early stopping criteria. For baseline models (IDs 1–4), training consistently converged earlier and was therefore terminated by early stopping at approximately 250 epochs. From ID 5 onward, models required longer training and were allowed to train for up to 350 epochs under the same early stopping protocol. This group includes the complete **Generator 1 + Discriminator 1** pipeline (IDs 5–12) as well as the multimodal configurations (IDs 13–14). For all experiments, training was stopped once validation performance saturated, ensuring a fair and consistent comparison across models.

IDs 5–12 employ **Generator 1**, which integrates temporal CNNs, super-resolution modules, and StyleGAN-inspired blocks, together with **Discriminator 1**, a multi-scale patch-based discriminator. IDs 13–14 use **Generator 2**, an extended architecture that incorporates temporal cross-frame attention to jointly predict future OCT frames and logMAR visual acuity.

Performance metrics include SSIM and PSNR for image reconstruction quality, FID for distributional fidelity, and MAE/MSE for multimodal logMAR prediction accuracy. Three loss formulations are evaluated:

- **Loss_1_:** This baseline configuration employs a standard binary cross-entropy (BCE) loss for the discriminator (see Section 3.6.2). The generator is optimized primarily through a perceptual loss to capture high-level features, as detailed in Section 3.6.1.
- **Loss_2_:** This configuration introduces additional regularization for training stability and structural accuracy. The discriminator utilizes a combination of BCE and a gradient penalty (GP), as defined in Section 3.6.2. The generator loss is expanded to include perceptual, pixel-wise, adversarial, and Structural Similarity Index (SSIM) components to ensure both global consistency and local detail.
- **Loss_3_:** Our most comprehensive formulation integrates the Wasserstein distance into the adversarial framework. The discriminator is optimized using the composite loss $L_{D}$ defined in Equation (11), which combines BCE, Wasserstein loss, and GP. Correspondingly, the generator is trained using the total loss $L_{G}$ defined in Equation (10), incorporating the Wasserstein-based adversarial signal alongside perceptual, pixel, and SSIM losses. Detailed formulations for these components are provided in Section 3.6.1 and Section 3.6.2.

The best overall performance is achieved by the multimodal temporal attention model (ID 14), trained for up to 350 epochs, which attains an SSIM of 0.9264 and a PSNR of 38.1.

- **SSIM**: Measures perceptual and structural similarity between predicted and ground-truth OCT images.
- **PSNR**: Reflects pixel-level fidelity and noise robustness.
- **MAE** and **MSE**: Capture absolute and squared errors in logMAR prediction (only applicable in multimodal models).

*Baseline Performance*

A transformer-based model with positional encoding and perceptual loss (row 1) yields poor visual quality (**SSIM = 0.5432**, **PSNR = 0.1314**), underscoring the limitations of using only static image encoding and no temporal modeling.

*Effect of Temporal Modeling and StyleGAN Blocks*

Introducing temporal CNNs and super-resolution modules (Generator_1) markedly improves performance. For example,

- v1, v2 → v3 improves to **SSIM = 0.7938**, **PSNR = 0.2931**;
- v1, v2, v3, v4 → v5 achieves **SSIM = 0.8611**, indicating the benefit of longer temporal windows.

*Multimodal Forecasting with Generator_2*

We began our experiments with a baseline generator architecture (Models ID 1–4), establishing reference performance for single-visit and simple paired-input forecasting. Building on these results, we adopted the enhanced **Generator_2** design with temporal attention and multimodal fusion. This model was evaluated using two-pair, three-pair, and four-pair input sequences to predict the next OCT frame and its corresponding logMAR value.

Due to dataset limitations—specifically the inconsistency of longer longitudinal sequences—we focused on the most reliable configurations: **two-pair** and **two-pair** inputs. Among all tested settings, the three-pair multimodal configuration produced the best overall performance, as summarized in Table 5.

The final Generator_2 model (three-pair input) achieves the following:

- **SSIM = 0.9264 ± 0.006**, **PSNR = 38.1 ± 0.9**;
- **MAE = 0.052**, **MSE = 0.0058**.

These results confirm the synergy of multimodal learning and dynamic attention-driven temporal encoding. The improved logMAR prediction further highlights the model’s ability to forecast functional vision outcomes alongside structural OCT progression, even with limited longitudinal data.

### 4.6. Biomarker Classification Results

In addition to anatomical and functional forecasting, we add a classification model at the end to predict 16 clinically relevant retinal biomarkers from the generated OCT scans using a pretrained EfficientNet-B0 classifier. The results for these predicted biomarkers are summarized and discussed in Table 6. Quantitative evaluation on the OLIVES dataset demonstrates strong performance despite class imbalance. Using focal loss during training, the classifier achieved a macro-averaged **F1 score of 0.81**, with per-class F1 scores ranging from 0.72 (IR hemorrhages) to 0.89 (subretinal fluid).

### 4.7. Comparison with Prior Longitudinal Forecasting Models

Several prior studies have explored generative and predictive modeling of disease progression using longitudinal medical data. Notably, the Sequence-Aware Diffusion Model (SADM) [15] and the GRAPE dataset [16] serve as representative efforts in volumetric image generation and functional outcome prediction, respectively. In this section, we highlight how our proposed multimodal GAN framework advances beyond these approaches in terms of forecasting capacity, multimodal fusion, and clinical utility.

*Comparison with MT-DENet*

Both MT-DENet [19] and the proposed method are evaluated on the OLIVES/TREX-DME dataset and adopt GAN-based architectures for longitudinal OCT forecasting. However, the reported SSIM and PSNR values are obtained under different spatial evaluation protocols.

MT-DENet follows a lesion-centric formulation and evaluates prediction quality over the entire post-therapy OCT scan, which includes peripheral regions exhibiting substantial anatomical variability and heterogeneous treatment response. In contrast, our approach is function-centric and evaluates prediction fidelity within a fovea-centered region of interest, motivated by its direct clinical relevance to visual acuity (logMAR/BCVA). The foveal region exhibits comparatively lower structural variability and is the primary determinant of functional outcome in DME.

Consequently, SSIM and PSNR values reported for the proposed method are not directly comparable in an absolute numerical sense to full-field evaluations reported by MT-DENet. The substantially higher values observed in Table 7 therefore reflect differences in evaluation scope and clinical focus rather than a direct one-to-one performance comparison under identical conditions. The comparison is intended to contextualize methodological differences and highlight the impact of region-specific evaluation strategies in longitudinal OCT prediction.

*Summary*

Our multimodal GAN approach introduces a novel integration of spatiotemporal generation and clinical forecasting. By combining StyleGAN blocks, temporal attention, and a DeepShallow LSTM, our model offers both fine-grained anatomical realism and accurate clinical predictions, surpassing the scope of prior methods.

### 4.8. Loss Function Ablation

The loss functions used in generator training significantly affect outcome quality:

- **Loss_1** (BCE + perceptual loss): Produces sharp but occasionally unstable predictions.
- **Loss_2** (adds gradient penalty, SSIM, pixel-wise terms): Yields improved convergence and sharper details.
- **Loss_3** (adds Wasserstein term): Stabilizes adversarial training and maximizes perceptual quality.

Overall, Loss_3 in combination with Generator_2 produces the highest visual and functional fidelity.

## 5. Discussion

This study introduces a multimodal generative forecasting framework capable of predicting future OCT anatomy, visual acuity (logMAR), and biomarker evolution using longitudinal ophthalmic data. By integrating temporal attention, StyleGAN-inspired blocks, super-resolution modules, and a DeepShallow LSTM, the model jointly captures both structural and functional disease progression. The results demonstrate that this multimodal formulation substantially outperforms image-only and scalar-only baselines, underscoring the value of combining imaging and clinical time-series information for ophthalmic prognosis.

### 5.1. Interpretation of Anatomical and Functional Forecasting

The proposed architecture achieves high-fidelity anatomical predictions, with SSIM values reaching 0.9264 and PSNR exceeding 38 dB. These metrics indicate that the model effectively preserves retinal layer continuity, foveal contour, and fluid-related microstructures—elements that are essential for monitoring diabetic macular edema (DME) and other retinal diseases. The incorporation of temporal attention enables the model to prioritize the most diagnostically informative visits, improving its ability to reconstruct subtle disease trajectories and distinguishing it from conventional recurrent or convolutional temporal encoders.

The multimodal logMAR forecasting component also performs strongly, achieving a mean absolute error (MAE) of 0.052, which is well within the typical ±0.1 logMAR test–retest variability. Importantly, the forecasts do not simply regress toward mean values; instead, they maintain the directionality of visual function changes across visits. Using the Winner–Stabilizer–Loser framework, the model demonstrates robust trend-aware predictions, aligning with clinically observed improvements, stabilizations, and deteriorations.

### 5.2. Comparison with Prior Longitudinal Imaging Models

Compared to diffusion-based approaches such as SADM, which achieves SSIM values around 0.85 on cardiac MRI, the proposed GAN-based framework offers superior structural fidelity while enabling faster training and inference. Models associated with the GRAPE dataset primarily focus on visual field progression without generating future anatomical images, whereas our method jointly synthesizes future OCT frames and functional outcomes, providing a more comprehensive understanding of disease evolution.

The combination of StyleGAN blocks, super-resolution modules, and adversarial supervision is particularly effective for ophthalmic imaging, where fine structural details and layer boundaries must be preserved. By contrast, transformer-only or CNN-only baselines in earlier experiments produce lower SSIM and PSNR values, highlighting the importance of the proposed multimodal temporal architecture.

### 5.3. Clinical Relevance

The forecasting capability of the model has direct clinical implications:

- **Proactive monitoring**: Predicting OCT structural changes in advance enables earlier detection of disease progression.
- **Personalized therapy planning**: Forecasted OCT and logMAR trajectories offer insights into expected responses to treatment, particularly anti-VEGF regimens.
- **Digital retinal twins**: The model supports individualized longitudinal simulations, enhancing population-level modeling and clinical trial design.

The strong biomarker detection performance on generated OCTs (macro-F1 = 0.81) further validates the anatomical realism of the synthesized images. These findings support the utility of such models in decision-support systems for retinal disease management.

### 5.4. Limitations

Several limitations should be considered:

- The OLIVES dataset, although rich in longitudinal information, includes only 96 eyes, which is modest for training deep generative models.
- Qualitative assessment was centered on the middle B-scan; full 3D volumetric evaluation remains to be explored.
- Biomarker annotations are available only for the first and last visits, limiting longitudinal biomarker supervision.
- Treatment events (e.g., anti-VEGF injections) are not explicitly encoded, despite their influence on disease trajectories.
- Multi-step autoregressive rollouts may accumulate error over longer prediction horizons.

Addressing these limitations will enhance generalizability and support deployment in clinical environments.

### 5.5. Future Work

Future research directions include the following:

- Extending the proposed generator to full 3D volumetric OCT forecasting, enabling spatially consistent prediction across entire retinal volumes.
- Incorporating detailed treatment history and dosage information to support treatment-aware and patient-specific forecasting.
- Exploring hybrid GAN–diffusion frameworks to improve global structural coherence while preserving fine-grained anatomical details.
- Applying interpretability tools such as attention visualization and saliency maps to enhance clinical trust and model transparency.
- Performing cross-dataset validation on additional longitudinal ophthalmic datasets to assess robustness and generalizability beyond the OLIVES cohort.
- While the present analysis does not explicitly model temporal treatment dynamics, future studies should incorporate time-dependent effects associated with the three dosing regimens—fixed, PRN (pro re nata, *as needed*), and treat-and-extend—to more accurately capture their differential impact on therapeutic outcomes.
- Future work should also directly compare the temporal effectiveness of anti-VEGF therapy with corticosteroid-based treatments, clarifying their relative benefits across distinct disease activity patterns and patient subgroups.

In addition, future studies will adapt and evaluate state-of-the-art longitudinal OCT forecasting and retinal modeling approaches under the unified evaluation protocol presented in Table 7. Rather than relying on heterogeneous experimental settings reported in the prior literature, these models will be re-implemented or re-trained to be conform to our fovea-centered forecasting framework, standardized preprocessing, and clinically motivated evaluation metrics. This controlled benchmarking will enable a fair and rigorous assessment of relative model performance and further validate the effectiveness of the proposed methodology.

To advance the field more broadly, we encourage the medical and research community to generate and share similarly detailed real-world longitudinal datasets—beyond those currently available in the OLIVES dataset—to support more comprehensive analyses of treatment patterns, temporal dynamics, and comparative therapeutic outcomes across diverse patient populations.

## Figures and Tables

**Table 2:** Detailed modality breakdown and annotations in the OLIVES dataset. Here, $N_{P}$ denotes the number of visits for a given eye. The total number of fundus visits across all eyes is $\sum N_{P}$ = 1268 (average ∼16 visits per eye). Not all modalities are acquired at every visit; in particular, OCT volumes are available for additional visits, resulting in a higher total number of OCT scans.

Modality	Per Visit	Per Eye	Total	Type	Notes
OCT (3D)	49 B-scans	$N_{P}^{\mathrm{OCT}}\times 49$	78,189	3D volume	One OCT volume per visit; OCT available for $N_{P}^{\mathrm{OCT}}$ = 1595 visits
Fundus (NIR)	1 image	$N_{P}$	1268	2D image	One fundus image per visit
Clinical Labels	4 labels	$N_{P}^{\mathrm{OCT}}\times 4$	6380	Tabular	BCVA, CST, patient ID, eye ID (recorded at OCT visits)
Biomarkers	16	16	1536	Vector	Annotated per eye at baseline and final visit
Disease Label	—	1	96	Category	DR or DME per eye (static)
Time Series	—	∼16	—	Mixed	Longitudinal follow-up over ∼2 years

**Table 3:** Winner–Stabilizer–Loser counts for each patient at different Δ thresholds for GT and Predicted logMAR.

Patient	Class	0.01	0.02	0.03	0.04	0.05	0.06	0.07	0.08	0.09	0.10
**Ground Truth (GT)**											
201	Winner	5	6	7	8	8	8	8	8	8	8
201	Stabilizer	10	11	12	12	12	12	12	12	12	12
201	Loser	5	3	1	0	0	0	0	0	0	0
203	Winner	3	4	5	6	6	6	6	7	7	7
203	Stabilizer	9	10	11	12	12	12	12	12	12	12
203	Loser	4	3	2	1	1	1	1	1	1	1
232	Winner	2	2	3	3	2	1	1	1	1	1
232	Stabilizer	8	10	14	18	21	22	22	23	23	23
232	Loser	13	11	6	2	0	0	0	0	0	0
234	Winner	3	3	4	5	5	6	6	6	6	6
234	Stabilizer	8	9	10	11	11	12	12	12	12	12
234	Loser	6	5	4	2	1	0	0	0	0	0
**Predicted (Pred)**											
201	Winner	6	7	7	8	8	8	8	8	8	8
201	Stabilizer	9	10	11	11	12	12	12	12	12	12
201	Loser	5	4	2	1	0	0	0	0	0	0
203	Winner	3	4	5	5	6	6	6	6	6	6
203	Stabilizer	9	10	11	12	12	12	12	12	12	12
203	Loser	4	3	2	1	1	1	1	1	1	1
232	Winner	3	3	3	3	2	1	1	1	1	1
232	Stabilizer	8	9	13	17	20	21	21	22	22	22
232	Loser	12	10	6	2	0	0	0	0	0	0
234	Winner	3	3	4	5	5	6	6	6	6	6
234	Stabilizer	8	9	10	11	11	12	12	12	12	12
234	Loser	6	5	4	2	1	0	0	0	0	0

**Table 4:** Per-patient performance metrics using macro, micro, and weighted averaging across the three classes—Winner (W), Stabilizer (S), and Loser (L)—computed using the threshold Δ=0.05.

Patient	Prec (Ma)	Rec (Ma)	F1 (Ma)	Prec (Mi)	Rec (Mi)	F1 (Mi)	Prec (Wt)	Rec (Wt)	F1 (Wt)
**Overall**	0.8261	0.8552	0.8382	0.8376	0.8376	0.8376	0.8420	0.8376	0.8376
**201**	0.8889	0.9744	0.9200	0.9500	0.9500	0.9500	0.9667	0.9500	0.9540
**203**	0.9444	0.9333	0.9327	0.9231	0.9231	0.9231	0.9359	0.9231	0.9223
**204**	0.7500	0.7714	0.7453	0.7391	0.7391	0.7391	0.7536	0.7391	0.7327
**232**	0.8287	0.8444	0.8287	0.8261	0.8261	0.8261	0.8345	0.8261	0.8219

**Table 5:** Performance comparison across model configurations using SSIM, PSNR, and FID. MAE and MSE apply only to the multimodal models (IDs 13–14), which jointly predict OCT frames and logMAR scores. Model ID-13 achieves MAE = 0.078 and MSE = 0.0066, while Model ID-14 achieves MAE = 0.052 and MSE = 0.0058.

ID	Input	Output	SSIM	PSNR	FID
1	v1, v2	v3	0.5432 ± 0.012	21.3 ± 1.7	101.3
2	v1, v2	v3	0.7654 ± 0.009	27.8 ± 1.2	55.6
3	v1, v2	v3	0.7938 ± 0.010	29.3 ± 1.1	47.2
4	v1, v2, v3	v4	0.8232 ± 0.011	30.1 ± 1.0	38.8
5	v1, v2, v3	v4	0.8411 ± 0.010	31.3 ± 1.0	33.5
6	v1, v2, v3, v4	v5	0.8611 ± 0.009	32.8 ± 1.1	27.9
7	v2, v3, v4, v5	v6	0.8612 ± 0.010	32.9 ± 1.2	27.6
8	v3, v4, v5	v6	0.8727 ± 0.009	33.0 ± 1.1	25.8
9	v4, v5, v6	v7	0.8841 ± 0.008	33.5 ± 1.1	23.9
10	v5, v6, v7	v8	0.8732 ± 0.009	33.8 ± 1.0	24.7
11	v6, v7, v8	v9	0.8797 ± 0.008	34.1 ± 1.0	24.1
12	v7, v8, v9	v10	0.8864 ± 0.009	35.1 ± 1.0	22.8
13	v1–v9 (two pairs)	v3–v10	0.8992 ± 0.007	36.2 ± 1.0	13.8
14	v1–v9 (three pairs)	v3–v10	**0.9264 ± 0.006**	**38.1 ± 0.9**	**11.9**

**Table 6:** Per-class biomarker classification performance (precision, recall, F1) for EfficientNet-B0 on the OLIVES dataset. Macro-F1 = 0.81.

Biomarker	Precision	Recall	F1 Score
Atrophy/thinning of retinal layers	0.82	0.79	0.80
Disruption of ellipsoid zone (EZ)	0.83	0.84	0.83
Disorganization of retinal inner layers (DRIL)	0.81	0.75	0.78
Intraretinal hemorrhages (IR)	0.70	0.74	0.72
Intraretinal hyperreflective foci (IRHRF)	0.76	0.80	0.78
Partially attached vitreous face (PAVF)	0.82	0.81	0.81
Fully attached vitreous face (FAVF)	0.85	0.83	0.84
Preretinal tissue/hemorrhage	0.77	0.79	0.78
Vitreous debris (VD)	0.80	0.78	0.79
Vitreomacular traction (VMT)	0.74	0.76	0.75
Diffuse retinal thickening/macular edema (DRT/ME)	0.86	0.85	0.85
Intraretinal fluid (IRF)	0.90	0.87	0.89
Subretinal fluid (SRF)	0.91	0.88	0.89
RPE disruption	0.83	0.82	0.83
Serous PED	0.76	0.78	0.77
Subretinal hyperreflective material (SHRM)	0.84	0.85	0.84

**Table 7:** Quantitative comparison of SSIM and PSNR with state-of-the-art longitudinal OCT prediction methods on the OLIVES/TREX-DME dataset. *Note: SSIM and PSNR values for prior methods are reported as published and are computed over full-field OCT scans, whereas results for our proposed method are evaluated within a fovea-centered region of interest*.

Method	SSIM ↑	PSNR ↑
AttenGAN	0.294±0.012	20.175±0.691
Pix2Pix	0.297±0.010	20.021±0.441
Atten2Angio	0.302±0.009	20.085±0.832
I2I-Mamba	0.325±0.014	20.578±0.331
SHENet (variant 1)	0.322±0.018	20.257±0.446
SHENet (variant 2)	0.329±0.013	20.507±0.305
MTDNet (variant 1)	0.331±0.015	20.460±0.344
MTDNet (variant 2)	0.333±0.010	20.702±0.373
MTDNet (best)	0.337±0.011	20.937±0.385
Ours (ID 13)	0.8992±0.007	36.2±1.0
Ours (ID 14)	0.9264±0.006	38.1±0.9

## Data Availability

We gratefully thank the authors of OLIVES [20] for providing us with their OCT dataset.

## Abbreviations

The following abbreviations are used in this manuscript:

CST	Central Subfield Thickness
logMAR	Logarithm of the Minimum Angle of Resolution
BCVA	Best-Corrected Visual Acuity
Eye ID	Eye Identity
EZ	Ellipsoid Zone
DRIL	Disruption of the Retinal Inner Layers
IR	Intraretinal
IRHRF	Intraretinal Hyperreflective Foci
PAVF	Partially Attached Vitreous Face
FAVF	Fully Attached Vitreous Face
VMT	Vitreomacular Traction
DRT/ME	Diffuse Retinal Thickening or Macular Edema
IRF	Intraretinal Fluid
SRF	Subretinal Fluid
RPE	Retinal Pigment Epithelium
PED	Pigment Epithelial Detachment
SHRM	Subretinal Hyperreflective Material
DR	Diabetic Retinopathy
DME	Diabetic Macular Edema
CI-DME	Center-Involved Diabetic Macular Edema
PDR	Proliferative Diabetic Retinopathy
NPDR	Non-Proliferative Diabetic Retinopathy
OCT	Optical Coherence Tomography
MAE	Mean Absolute Error
MSE	Mean Squared Error
PSNR	Peak Signal-to-Noise Ratio
SSIM	Structural Similarity Index Measure
BCE	Binary Cross-Entropy
GP	Gradient Penalty
AMD	Age-related Macular Degeneration
CNV	Choroidal Neovascularization
VEGF	Vascular Endothelial Growth Factor
ETDRS	Early Treatment Diabetic Retinopathy Study
OLIVES	Ophthalmic Labels for Investigating Visual Eye Semantics
FID	Fréchet Inception Distance
SVM	Support Vector Machine
RF	Random Forest
MLP	Extreme Learning Machine
ELM	Multi-Layer Perceptron
LR	Linear Regression
